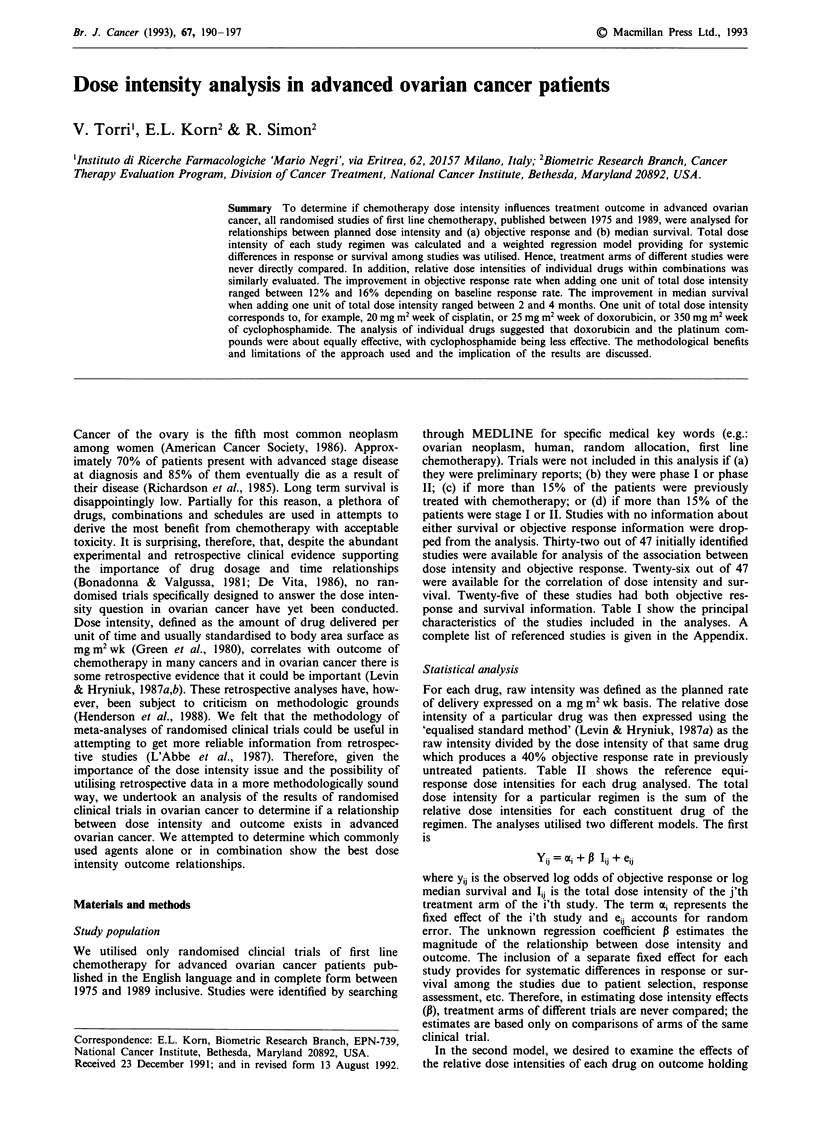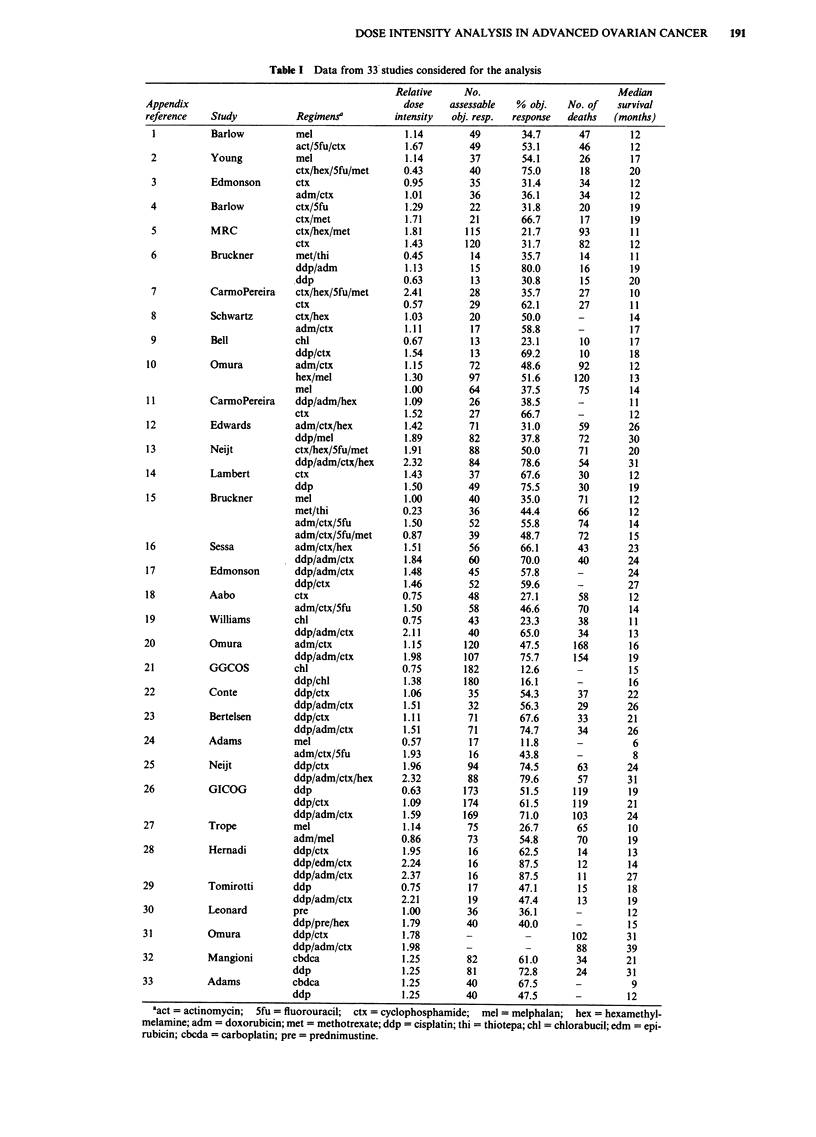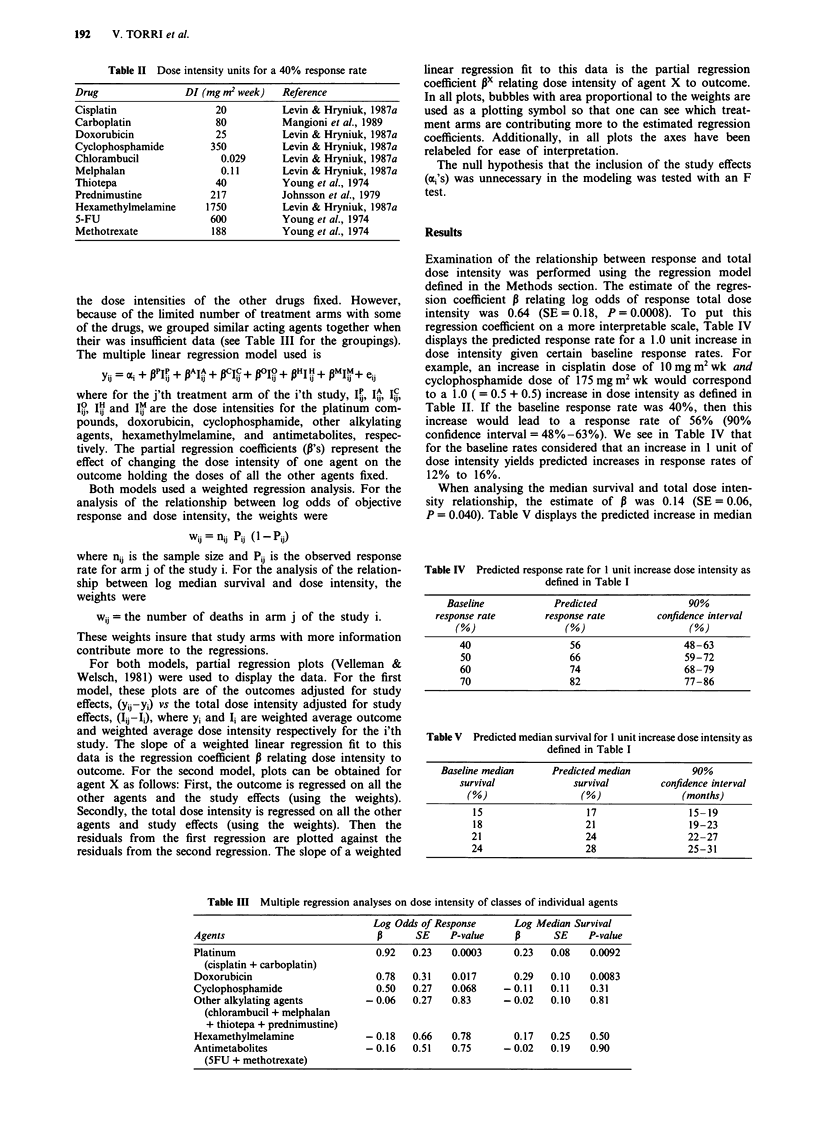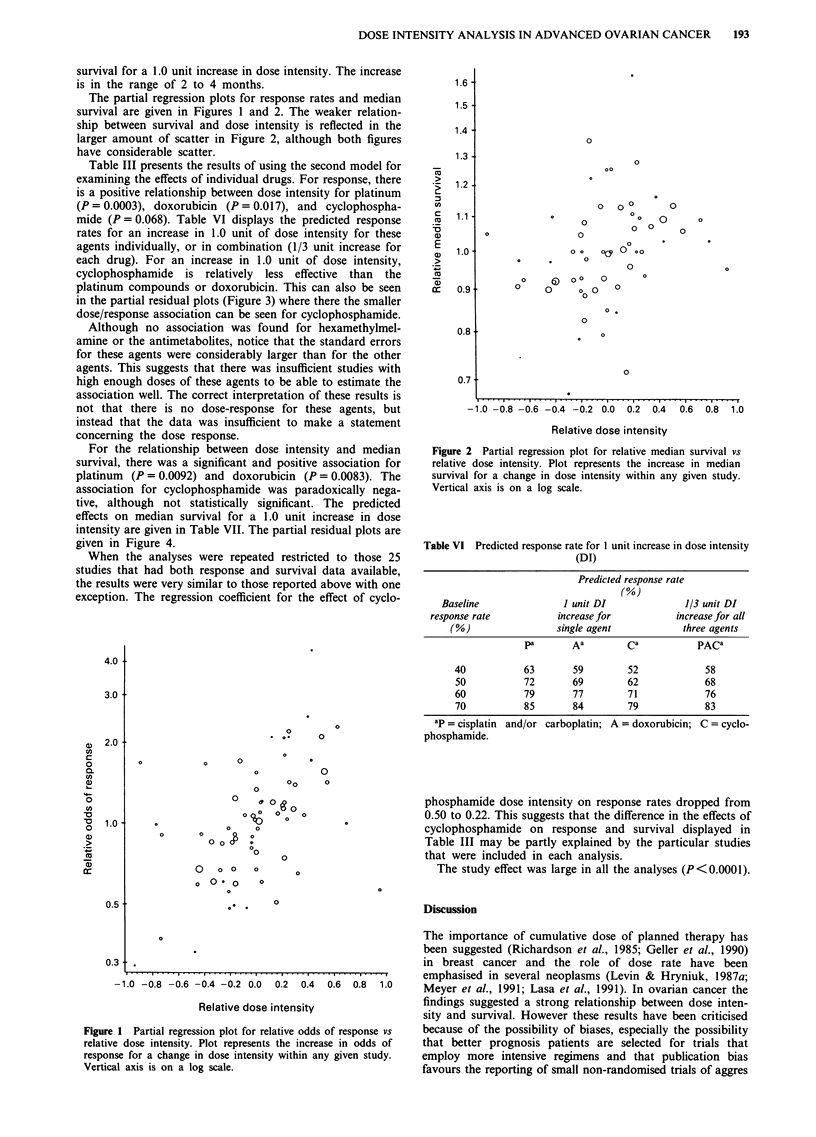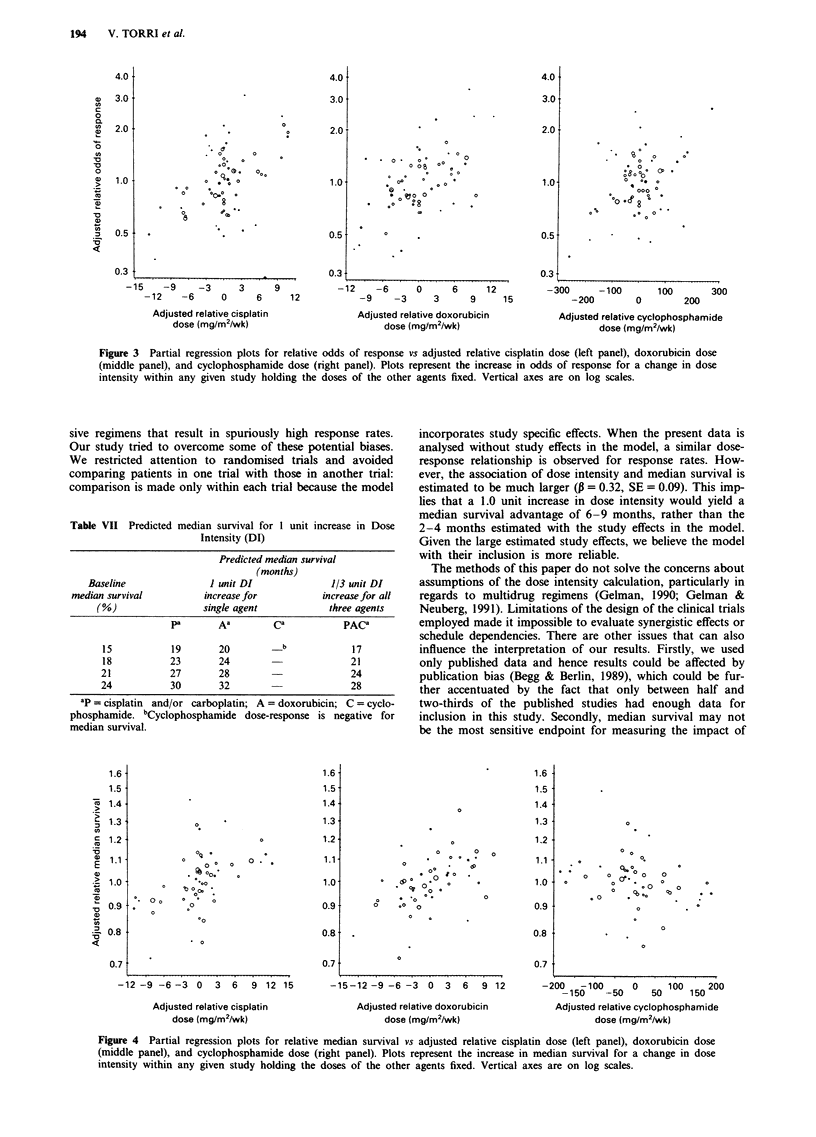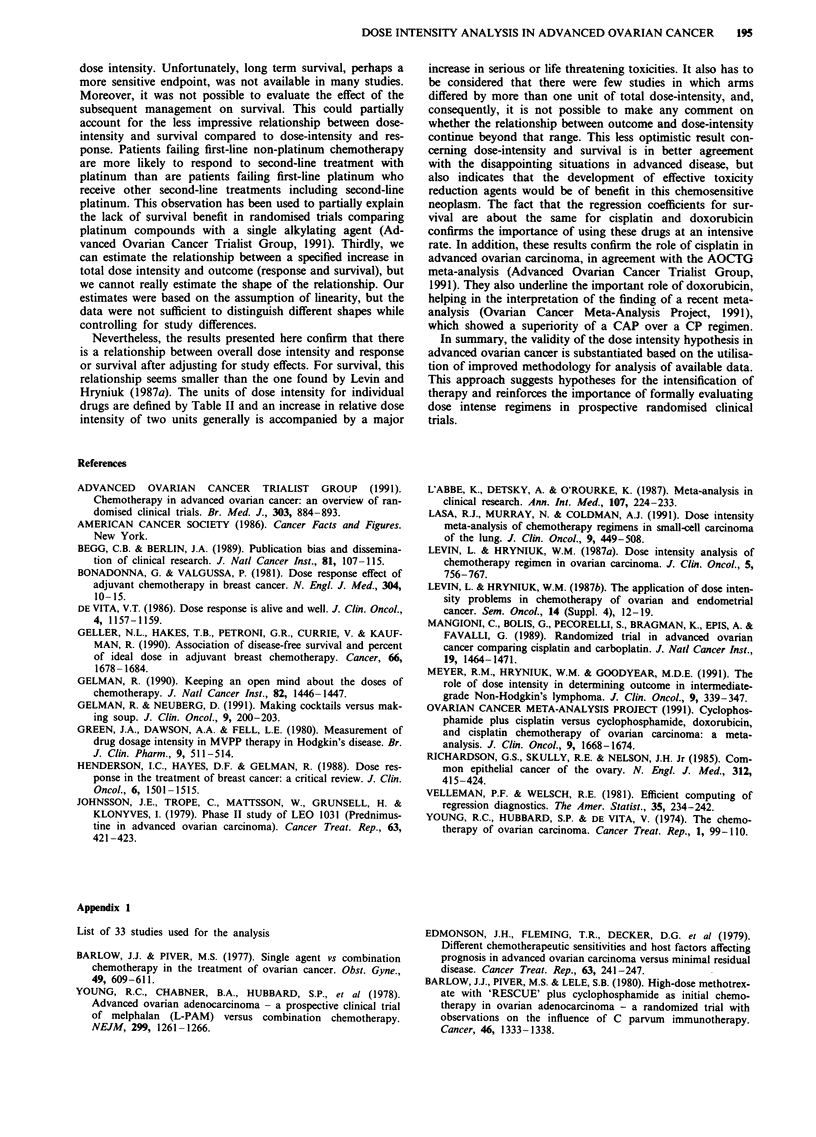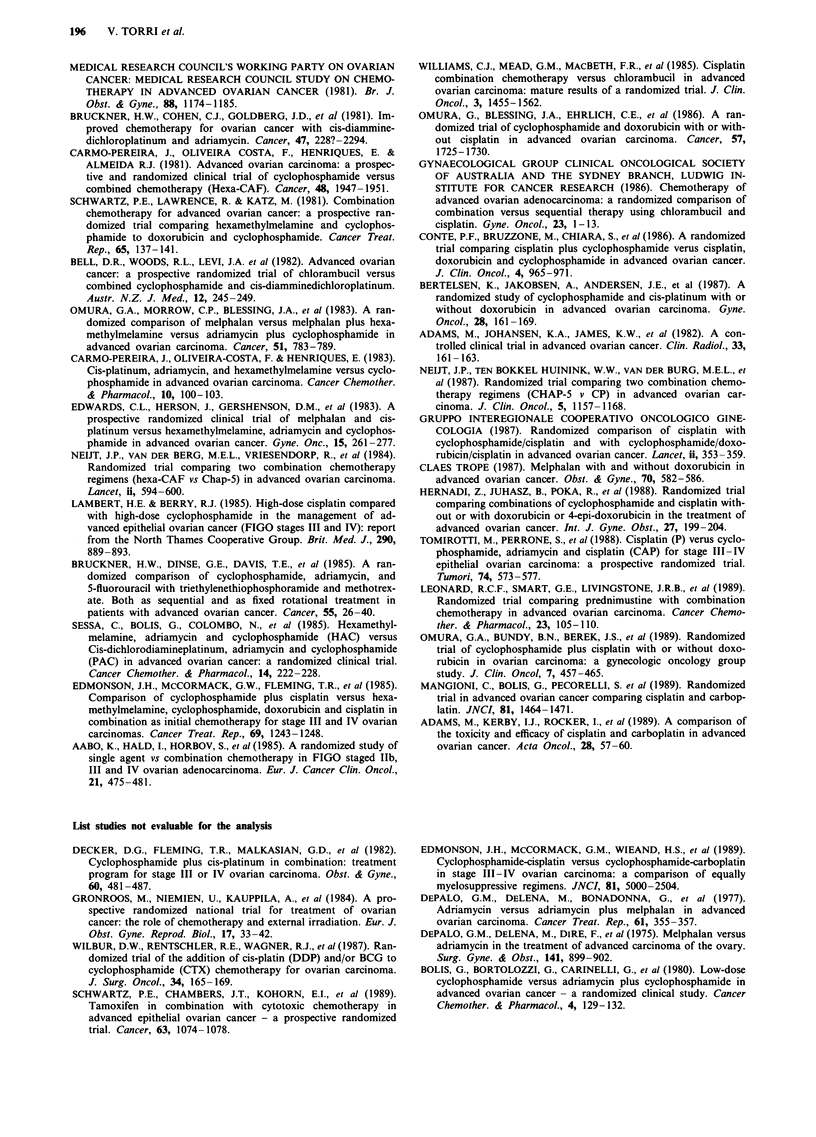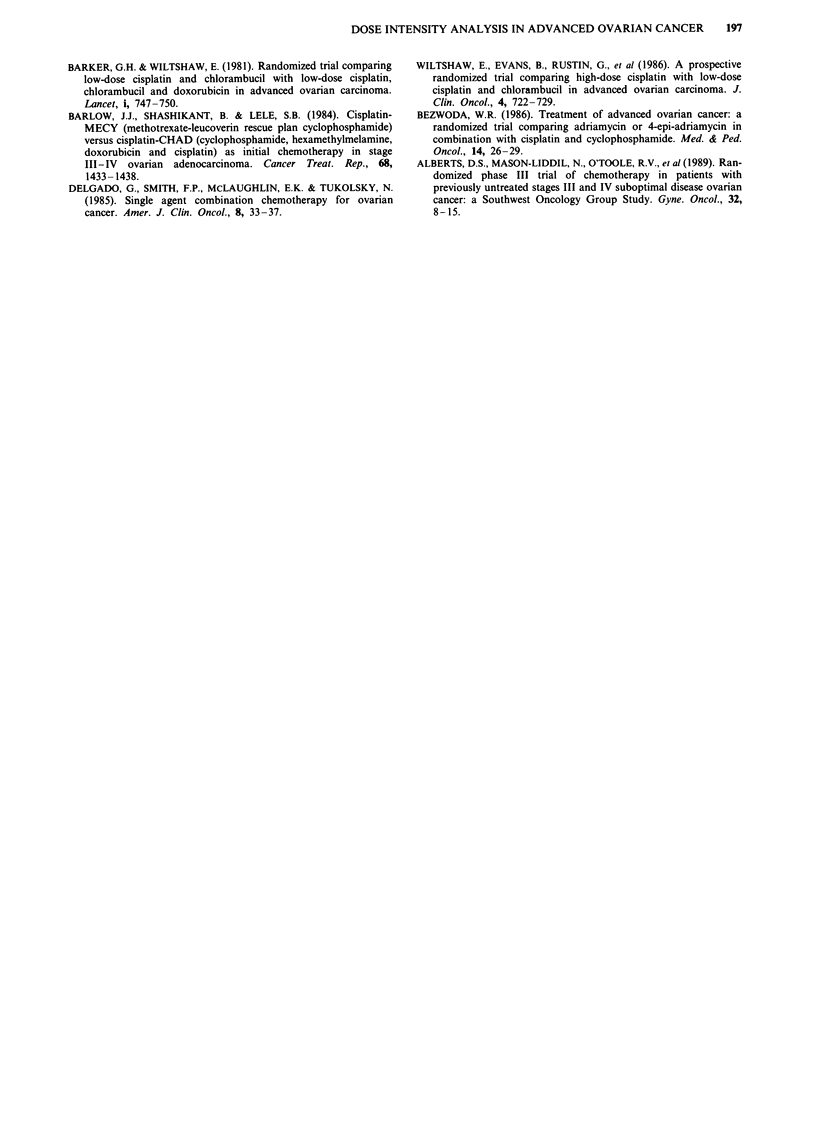# Dose intensity analysis in advanced ovarian cancer patients.

**DOI:** 10.1038/bjc.1993.33

**Published:** 1993-01

**Authors:** V. Torri, E. L. Korn, R. Simon

**Affiliations:** Instituto di Ricerche Farmacologiche Mario Negri, Milano, Italy.

## Abstract

To determine if chemotherapy dose intensity influences treatment outcome in advanced ovarian cancer, all randomised studies of first line chemotherapy, published between 1975 and 1989, were analysed for relationships between planned dose intensity and (a) objective response and (b) median survival. Total dose intensity of each study regimen was calculated and a weighted regression model providing for systemic differences in response or survival among studies was utilised. Hence, treatment arms of different studies were never directly compared. In addition, relative dose intensities of individual drugs within combinations was similarly evaluated. The improvement in objective response rate when adding one unit of total dose intensity ranged between 12% and 16% depending on baseline response rate. The improvement in median survival when adding one unit of total dose intensity ranged between 2 and 4 months. One unit of total dose intensity corresponds to, for example, 20 mg m2 week of cisplatin, or 25 mg m2 week of doxorubicin, or 350 mg m2 week of cyclophosphamide. The analysis of individual drugs suggested that doxorubicin and the platinum compounds were about equally effective, with cyclophosphamide being less effective. The methodological benefits and limitations of the approach used and the implication of the results are discussed.


*Br. J. Cancer* (1993), **67**, 190–197 © Macmillan Press Ltd., 1993

### Dose intensity analysis in advanced ovarian cancer patients

V. Torri[1], E.L. Korn[2] & R. Simon[2]

[1]*Instituto di Ricerche Farmacologiche 'Mario Negri', via Eritrea, 62, 20157 Milano, Italy;* [2]*Biometric Research Branch, Cancer Therapy Evaluation Program, Division of Cancer Treatment, National Cancer Institute, Bethesda, Maryland 20892, USA.*

**Summary** To determine if chemotherapy dose intensity influences treatment outcome in advanced ovarian cancer, all randomised studies of first line chemotherapy, published between 1975 and 1989, were analysed for relationships between planned dose intensity and (a) objective response and (b) median survival. Total dose intensity of each study regimen was calculated and a weighted regression model providing for systemic differences in response or survival among studies was utilised. Hence, treatment arms of different studies were never directly compared. In addition, relative dose intensities of individual drugs within combinations was similarly evaluated. The improvement in objective response rate when adding one unit of total dose intensity ranged between 12% and 16% depending on baseline response rate. The improvement in median survival when adding one unit of total dose intensity ranged between 2 and 4 months. One unit of total dose intensity corresponds to, for example, 20 mg m$^2$ week of cisplatin, or 25 mg m$^2$ week of doxorubicin, or 350 mg m$^2$ week of cyclophosphamide. The analysis of individual drugs suggested that doxorubicin and the platinum compounds were about equally effective, with cyclophosphamide being less effective. The methodological benefits and limitations of the approach used and the implication of the results are discussed.

Cancer of the ovary is the fifth most common neoplasm among women (American Cancer Society, 1986). Approximately 70% of patients present with advanced stage disease at diagnosis and 85% of them eventually die as a result of their disease (Richardson *et al.*, 1985). Long term survival is disappointingly low. Partially for this reason, a plethora of drugs, combinations and schedules are used in attempts to derive the most benefit from chemotherapy with acceptable toxicity. It is surprising, therefore, that, despite the abundant experimental and retrospective clinical evidence supporting the importance of drug dosage and time relationships (Bonadonna & Valgussa, 1981; De Vita, 1986), no randomised trials specifically designed to answer the dose intensity question in ovarian cancer have yet been conducted. Dose intensity, defined as the amount of drug delivered per unit of time and usually standardised to body area surface as mg m$^2$ wk (Green *et al.*, 1980), correlates with outcome of chemotherapy in many cancers and in ovarian cancer there is some retrospective evidence that it could be important (Levin & Hryniuk, 1987*a*,*b*). These retrospective analyses have, however, been subject to criticism on methodologic grounds (Henderson *et al.*, 1988). We felt that the methodology of meta-analyses of randomised clinical trials could be useful in attempting to get more reliable information from retrospective studies (L'Abbe *et al.*, 1987). Therefore, given the importance of the dose intensity issue and the possibility of utilising retrospective data in a more methodologically sound way, we undertook an analysis of the results of randomised clinical trials in ovarian cancer to determine if a relationship between dose intensity and outcome exists in advanced ovarian cancer. We attempted to determine which commonly used agents alone or in combination show the best dose intensity outcome relationships.

### Materials and methods

### *Study population*

We utilised only randomised clincial trials of first line chemotherapy for advanced ovarian cancer patients published in the English language and in complete form between 1975 and 1989 inclusive. Studies were identified by searching through MEDLINE for specific medical key words (e.g.: ovarian neoplasm, human, random allocation, first line chemotherapy). Trials were not included in this analysis if (a) they were preliminary reports; (b) they were phase I or phase II; (c) if more than 15% of the patients were previously treated with chemotherapy; or (d) if more than 15% of the patients were stage I or II. Studies with no information about either survival or objective response information were dropped from the analysis. Thirty-two out of 47 initially identified studies were available for analysis of the association between dose intensity and objective response. Twenty-six out of 47 were available for the correlation of dose intensity and survival. Twenty-five of these studies had both objective response and survival information. Table I show the principal characteristics of the studies included in the analyses. A complete list of referenced studies is given in the Appendix.

### *Statistical analysis*

For each drug, raw intensity was defined as the planned rate of delivery expressed on a mg m$^2$ wk basis. The relative dose intensity of a particular drug was then expressed using the 'equalised standard method' (Levin & Hryniuk, 1987*a*) as the raw intensity divided by the dose intensity of that same drug which produces a 40% objective response rate in previously untreated patients. Table II shows the reference equi-response dose intensities for each drug analysed. The total dose intensity for a particular regimen is the sum of the relative dose intensities for each constituent drug of the regimen. The analyses utilised two different models. The first is

$$Y_{ij} = \alpha_i + \beta\ I_{ij} + e_{ij}$$

where $y_{ij}$ is the observed log odds of objective response or log median survival and $I_{ij}$ is the total dose intensity of the j'th treatment arm of the i'th study. The term $\alpha_i$ represents the fixed effect of the i'th study and $e_{ij}$ accounts for random error. The unknown regression coefficient $\beta$ estimates the magnitude of the relationship between dose intensity and outcome. The inclusion of a separate fixed effect for each study provides for systematic differences in response or survival among the studies due to patient selection, response assessment, etc. Therefore, in estimating dose intensity effects ($\beta$), treatment arms of different trials are never compared; the estimates are based only on comparisons of arms of the same clinical trial.

In the second model, we desired to examine the effects of the relative dose intensities of each drug on outcome holding

Correspondence: E.L. Korn, Biometric Research Branch, EPN-739, National Cancer Institute, Bethesda, Maryland 20892, USA.
Received 23 December 1991; and in revised form 13 August 1992.

**Table I** Data from 33 studies considered for the analysis

| *Appendix reference* | *Study* | *Regimens*[a] | *Relative dose intensity* | *No. assessable obj. resp.* | *% obj. response* | *No. of deaths* | *Median survival (months)* |
|---|---|---|---|---|---|---|---|
| 1 | Barlow | mel | 1.14 | 49 | 34.7 | 47 | 12 |
| | | act/5fu/ctx | 1.67 | 49 | 53.1 | 46 | 12 |
| 2 | Young | mel | 1.14 | 37 | 54.1 | 26 | 17 |
| | | ctx/hex/5fu/met | 0.43 | 40 | 75.0 | 18 | 20 |
| 3 | Edmonson | ctx | 0.95 | 35 | 31.4 | 34 | 12 |
| | | adm/ctx | 1.01 | 36 | 36.1 | 34 | 12 |
| 4 | Barlow | ctx/5fu | 1.29 | 22 | 31.8 | 20 | 19 |
| | | ctx/met | 1.71 | 21 | 66.7 | 17 | 19 |
| 5 | MRC | ctx/hex/met | 1.81 | 115 | 21.7 | 93 | 11 |
| | | ctx | 1.43 | 120 | 31.7 | 82 | 12 |
| 6 | Bruckner | met/thi | 0.45 | 14 | 35.7 | 14 | 11 |
| | | ddp/adm | 1.13 | 15 | 80.0 | 16 | 19 |
| | | ddp | 0.63 | 13 | 30.8 | 15 | 20 |
| 7 | CarmoPereira | ctx/hex/5fu/met | 2.41 | 28 | 35.7 | 27 | 10 |
| | | ctx | 0.57 | 29 | 62.1 | 27 | 11 |
| 8 | Schwartz | ctx/hex | 1.03 | 20 | 50.0 | – | 14 |
| | | adm/ctx | 1.11 | 17 | 58.8 | – | 17 |
| 9 | Bell | chl | 0.67 | 13 | 23.1 | 10 | 17 |
| | | ddp/ctx | 1.54 | 13 | 69.2 | 10 | 18 |
| 10 | Omura | adm/ctx | 1.15 | 72 | 48.6 | 92 | 12 |
| | | hex/mel | 1.30 | 97 | 51.6 | 120 | 13 |
| | | mel | 1.00 | 64 | 37.5 | 75 | 14 |
| 11 | CarmoPereira | ddp/adm/hex | 1.09 | 26 | 38.5 | – | 11 |
| | | ctx | 1.52 | 27 | 66.7 | – | 12 |
| 12 | Edwards | adm/ctx/hex | 1.42 | 71 | 31.0 | 59 | 26 |
| | | ddp/mel | 1.89 | 82 | 37.8 | 72 | 30 |
| 13 | Neijt | ctx/hex/5fu/met | 1.91 | 88 | 50.0 | 71 | 20 |
| | | ddp/adm/ctx/hex | 2.32 | 84 | 78.6 | 54 | 31 |
| 14 | Lambert | ctx | 1.43 | 37 | 67.6 | 30 | 12 |
| | | ddp | 1.50 | 49 | 75.5 | 30 | 19 |
| 15 | Bruckner | mel | 1.00 | 40 | 35.0 | 71 | 12 |
| | | met/thi | 0.23 | 36 | 44.4 | 66 | 12 |
| | | adm/ctx/5fu | 1.50 | 52 | 55.8 | 74 | 14 |
| | | adm/ctx/5fu/met | 0.87 | 39 | 48.7 | 72 | 15 |
| 16 | Sessa | adm/ctx/hex | 1.51 | 56 | 66.1 | 43 | 23 |
| | | ddp/adm/ctx | 1.84 | 60 | 70.0 | 40 | 24 |
| 17 | Edmonson | ddp/adm/ctx | 1.48 | 45 | 57.8 | – | 24 |
| | | ddp/ctx | 1.46 | 52 | 59.6 | – | 27 |
| 18 | Aabo | ctx | 0.75 | 48 | 27.1 | 58 | 12 |
| | | adm/ctx/5fu | 1.50 | 58 | 46.6 | 70 | 14 |
| 19 | Williams | chl | 0.75 | 43 | 23.3 | 38 | 11 |
| | | ddp/adm/ctx | 2.11 | 40 | 65.0 | 34 | 13 |
| 20 | Omura | adm/ctx | 1.15 | 120 | 47.5 | 168 | 16 |
| | | ddp/adm/ctx | 1.98 | 107 | 75.7 | 154 | 19 |
| 21 | GGCOS | chl | 0.75 | 182 | 12.6 | – | 15 |
| | | ddp/chl | 1.38 | 180 | 16.1 | – | 16 |
| 22 | Conte | ddp/ctx | 1.06 | 35 | 54.3 | 37 | 22 |
| | | ddp/adm/ctx | 1.51 | 32 | 56.3 | 29 | 26 |
| 23 | Bertelsen | ddp/ctx | 1.11 | 71 | 67.6 | 33 | 21 |
| | | ddp/adm/ctx | 1.51 | 71 | 74.7 | 34 | 26 |
| 24 | Adams | mel | 0.57 | 17 | 11.8 | – | 6 |
| | | adm/ctx/5fu | 1.93 | 16 | 43.8 | – | 8 |
| 25 | Neijt | ddp/ctx | 1.96 | 94 | 74.5 | 63 | 24 |
| | | ddp/adm/ctx/hex | 2.32 | 88 | 79.6 | 57 | 31 |
| 26 | GICOG | ddp | 0.63 | 173 | 51.5 | 119 | 19 |
| | | ddp/ctx | 1.09 | 174 | 61.5 | 119 | 21 |
| | | ddp/adm/ctx | 1.59 | 169 | 71.0 | 103 | 24 |
| 27 | Trope | mel | 1.14 | 75 | 26.7 | 65 | 10 |
| | | adm/mel | 0.86 | 73 | 54.8 | 70 | 19 |
| 28 | Hernadi | ddp/ctx | 1.95 | 16 | 62.5 | 14 | 13 |
| | | ddp/edm/ctx | 2.24 | 16 | 87.5 | 12 | 14 |
| | | ddp/adm/ctx | 2.37 | 16 | 87.5 | 11 | 27 |
| 29 | Tomirotti | ddp | 0.75 | 17 | 47.1 | 15 | 18 |
| | | ddp/adm/ctx | 2.21 | 19 | 47.4 | 13 | 19 |
| 30 | Leonard | pre | 1.00 | 36 | 36.1 | – | 12 |
| | | ddp/pre/hex | 1.79 | 40 | 40.0 | – | 15 |
| 31 | Omura | ddp/ctx | 1.78 | – | – | 102 | 31 |
| | | ddp/adm/ctx | 1.98 | – | – | 88 | 39 |
| 32 | Mangioni | cbdca | 1.25 | 82 | 61.0 | 34 | 21 |
| | | ddp | 1.25 | 81 | 72.8 | 24 | 31 |
| 33 | Adams | cbdca | 1.25 | 40 | 67.5 | – | 9 |
| | | ddp | 1.25 | 40 | 47.5 | – | 12 |

[a]act = actinomycin; 5fu = fluorouracil; ctx = cyclophosphamide; mel = melphalan; hex = hexamethylmelamine; adm = doxorubicin; met = methotrexate; ddp = cisplatin; thi = thiotepa; chl = chlorabucil; edm = epirubicin; cbcda = carboplatin; pre = prednimustine.

**Table II** Dose intensity units for a 40% response rate

| *Drug* | *DI (mg m² week)* | *Reference* |
|---|---|---|
| Cisplatin | 20 | Levin & Hryniuk, 1987*a* |
| Carboplatin | 80 | Mangioni *et al.*, 1989 |
| Doxorubicin | 25 | Levin & Hryniuk, 1987*a* |
| Cyclophosphamide | 350 | Levin & Hryniuk, 1987*a* |
| Chlorambucil | 0.029 | Levin & Hryniuk, 1987*a* |
| Melphalan | 0.11 | Levin & Hryniuk, 1987*a* |
| Thiotepa | 40 | Young *et al.*, 1974 |
| Prednimustine | 217 | Johnsson *et al.*, 1979 |
| Hexamethylmelamine | 1750 | Levin & Hryniuk, 1987*a* |
| 5-FU | 600 | Young *et al.*, 1974 |
| Methotrexate | 188 | Young *et al.*, 1974 |

the dose intensities of the other drugs fixed. However, because of the limited number of treatment arms with some of the drugs, we grouped similar acting agents together when their was insufficient data (see Table III for the groupings). The multiple linear regression model used is

$$y_{ij} = \alpha_i + \beta^P I^P_{ij} + \beta^A I^A_{ij} + \beta^C I^C_{ij} + \beta^O I^O_{ij} + \beta^H I^H_{ij} + \beta^M I^M_{ij} + e_{ij}$$

where for the j'th treatment arm of the i'th study, $I^P_{ij}$, $I^A_{ij}$, $I^C_{ij}$, $I^O_{ij}$, $I^H_{ij}$ and $I^M_{ij}$ are the dose intensities for the platinum compounds, doxorubicin, cyclophosphamide, other alkylating agents, hexamethylmelamine, and antimetabolites, respectively. The partial regression coefficients ($\beta$'s) represent the effect of changing the dose intensity of one agent on the outcome holding the doses of all the other agents fixed.

Both models used a weighted regression analysis. For the analysis of the relationship between log odds of objective response and dose intensity, the weights were

$$w_{ij} = n_{ij}\ P_{ij}\ (1 - P_{ij})$$

where $n_{ij}$ is the sample size and $P_{ij}$ is the observed response rate for arm j of the study i. For the analysis of the relationship between log median survival and dose intensity, the weights were

$w_{ij}$ = the number of deaths in arm j of the study i.

These weights insure that study arms with more information contribute more to the regressions.

For both models, partial regression plots (Velleman & Welsch, 1981) were used to display the data. For the first model, these plots are of the outcomes adjusted for study effects, $(y_{ij} - y_i)$ *vs* the total dose intensity adjusted for study effects, $(I_{ij} - I_i)$, where $y_i$ and $I_i$ are weighted average outcome and weighted average dose intensity respectively for the i'th study. The slope of a weighted linear regression fit to this data is the regression coefficient $\beta$ relating dose intensity to outcome. For the second model, plots can be obtained for agent X as follows: First, the outcome is regressed on all the other agents and the study effects (using the weights). Secondly, the total dose intensity is regressed on all the other agents and study effects (using the weights). Then the residuals from the first regression are plotted against the residuals from the second regression. The slope of a weighted linear regression fit to this data is the partial regression coefficient $\beta^X$ relating dose intensity of agent X to outcome. In all plots, bubbles with area proportional to the weights are used as a plotting symbol so that one can see which treatment arms are contributing more to the estimated regression coefficients. Additionally, in all plots the axes have been relabeled for ease of interpretation.

The null hypothesis that the inclusion of the study effects ($\alpha_i$'s) was unnecessary in the modeling was tested with an F test.

### Results

Examination of the relationship between response and total dose intensity was performed using the regression model defined in the Methods section. The estimate of the regression coefficient $\beta$ relating log odds of response total dose intensity was 0.64 (SE = 0.18, $P = 0.0008$). To put this regression coefficient on a more interpretable scale, Table IV displays the predicted response rate for a 1.0 unit increase in dose intensity given certain baseline response rates. For example, an increase in cisplatin dose of 10 mg m² wk *and* cyclophosphamide dose of 175 mg m² wk would correspond to a 1.0 ( = 0.5 + 0.5) increase in dose intensity as defined in Table II. If the baseline response rate was 40%, then this increase would lead to a response rate of 56% (90% confidence interval = 48%–63%). We see in Table IV that for the baseline rates considered that an increase in 1 unit of dose intensity yields predicted increases in response rates of 12% to 16%.

When analysing the median survival and total dose intensity relationship, the estimate of $\beta$ was 0.14 (SE = 0.06, $P = 0.040$). Table V displays the predicted increase in median

**Table IV** Predicted response rate for 1 unit increase dose intensity as defined in Table I

| *Baseline response rate (%)* | *Predicted response rate (%)* | *90% confidence interval (%)* |
|---|---|---|
| 40 | 56 | 48–63 |
| 50 | 66 | 59–72 |
| 60 | 74 | 68–79 |
| 70 | 82 | 77–86 |

**Table V** Predicted median survival for 1 unit increase dose intensity as defined in Table I

| *Baseline median survival (%)* | *Predicted median survival (%)* | *90% confidence interval (months)* |
|---|---|---|
| 15 | 17 | 15–19 |
| 18 | 21 | 19–23 |
| 21 | 24 | 22–27 |
| 24 | 28 | 25–31 |

**Table III** Multiple regression analyses on dose intensity of classes of individual agents

| | *Log Odds of Response* | | | *Log Median Survival* | | |
|---|---|---|---|---|---|---|
| *Agents* | $\beta$ | *SE* | *P-value* | $\beta$ | *SE* | *P-value* |
| Platinum (cisplatin + carboplatin) | 0.92 | 0.23 | 0.0003 | 0.23 | 0.08 | 0.0092 |
| Doxorubicin | 0.78 | 0.31 | 0.017 | 0.29 | 0.10 | 0.0083 |
| Cyclophosphamide | 0.50 | 0.27 | 0.068 | − 0.11 | 0.11 | 0.31 |
| Other alkylating agents (chlorambucil + melphalan + thiotepa + prednimustine) | − 0.06 | 0.27 | 0.83 | − 0.02 | 0.10 | 0.81 |
| Hexamethylmelamine | − 0.18 | 0.66 | 0.78 | 0.17 | 0.25 | 0.50 |
| Antimetabolites (5FU + methotrexate) | − 0.16 | 0.51 | 0.75 | − 0.02 | 0.19 | 0.90 |

survival for a 1.0 unit increase in dose intensity. The increase is in the range of 2 to 4 months.

The partial regression plots for response rates and median survival are given in Figures 1 and 2. The weaker relationship between survival and dose intensity is reflected in the larger amount of scatter in Figure 2, although both figures have considerable scatter.

Table III presents the results of using the second model for examining the effects of individual drugs. For response, there is a positive relationship between dose intensity for platinum ($P = 0.0003$), doxorubicin ($P = 0.017$), and cyclophosphamide ($P = 0.068$). Table VI displays the predicted response rates for an increase in 1.0 unit of dose intensity for these agents individually, or in combination (1/3 unit increase for each drug). For an increase in 1.0 unit of dose intensity, cyclophosphamide is relatively less effective than the platinum compounds or doxorubicin. This can also be seen in the partial residual plots (Figure 3) where there the smaller dose/response association can be seen for cyclophosphamide.

Although no association was found for hexamethylmelamine or the antimetabolites, notice that the standard errors for these agents were considerably larger than for the other agents. This suggests that there was insufficient studies with high enough doses of these agents to be able to estimate the association well. The correct interpretation of these results is not that there is no dose-response for these agents, but instead that the data was insufficient to make a statement concerning the dose response.

For the relationship between dose intensity and median survival, there was a significant and positive association for platinum ($P = 0.0092$) and doxorubicin ($P = 0.0083$). The association for cyclophosphamide was paradoxically negative, although not statistically significant. The predicted effects on median survival for a 1.0 unit increase in dose intensity are given in Table VII. The partial residual plots are given in Figure 4.

When the analyses were repeated restricted to those 25 studies that had both response and survival data available, the results were very similar to those reported above with one exception. The regression coefficient for the effect of cyclophosphamide dose intensity on response rates dropped from 0.50 to 0.22. This suggests that the difference in the effects of cyclophosphamide on response and survival displayed in Table III may be partly explained by the particular studies that were included in each analysis.

The study effect was large in all the analyses ($P < 0.0001$).

**Figure 1** Partial regression plot for relative odds of response *vs* relative dose intensity. Plot represents the increase in odds of response for a change in dose intensity within any given study. Vertical axis is on a log scale.

**Figure 2** Partial regression plot for relative median survival *vs* relative dose intensity. Plot represents the increase in median survival for a change in dose intensity within any given study. Vertical axis is on a log scale.

**Table VI** Predicted response rate for 1 unit increase in dose intensity (DI)

| *Baseline response rate (%)* | *Predicted response rate (%)* *1 unit DI increase for single agent* | | | *1/3 unit DI increase for all three agents* |
|---|---|---|---|---|
| | P[a] | A[a] | C[a] | PAC[a] |
| 40 | 63 | 59 | 52 | 58 |
| 50 | 72 | 69 | 62 | 68 |
| 60 | 79 | 77 | 71 | 76 |
| 70 | 85 | 84 | 79 | 83 |

[a]P = cisplatin and/or carboplatin; A = doxorubicin; C = cyclophosphamide.

### Discussion

The importance of cumulative dose of planned therapy has been suggested (Richardson *et al.*, 1985; Geller *et al.*, 1990) in breast cancer and the role of dose rate have been emphasised in several neoplasms (Levin & Hryniuk, 1987*a*; Meyer *et al.*, 1991; Lasa *et al.*, 1991). In ovarian cancer the findings suggested a strong relationship between dose intensity and survival. However these results have been criticised because of the possibility of biases, especially the possibility that better prognosis patients are selected for trials that employ more intensive regimens and that publication bias favours the reporting of small non-randomised trials of aggres

**Figure 3** Partial regression plots for relative odds of response *vs* adjusted relative cisplatin dose (left panel), doxorubicin dose (middle panel), and cyclophosphamide dose (right panel). Plots represent the increase in odds of response for a change in dose intensity within any given study holding the doses of the other agents fixed. Vertical axes are on log scales.

sive regimens that result in spuriously high response rates. Our study tried to overcome some of these potential biases. We restricted attention to randomised trials and avoided comparing patients in one trial with those in another trial: comparison is made only within each trial because the model incorporates study specific effects. When the present data is analysed without study effects in the model, a similar dose-response relationship is observed for response rates. However, the association of dose intensity and median survival is estimated to be much larger ($\beta = 0.32$, SE = 0.09). This implies that a 1.0 unit increase in dose intensity would yield a median survival advantage of 6–9 months, rather than the 2–4 months estimated with the study effects in the model. Given the large estimated study effects, we believe the model with their inclusion is more reliable.

**Table VII** Predicted median survival for 1 unit increase in Dose Intensity (DI)

| *Baseline median survival (%)* | *Predicted median survival (months)* | | | |
|---|---|---|---|---|
| | *1 unit DI increase for single agent* | | | *1/3 unit DI increase for all three agents* |
| | P[a] | A[a] | C[a] | PAC[a] |
| 15 | 19 | 20 | —[b] | 17 |
| 18 | 23 | 24 | — | 21 |
| 21 | 27 | 28 | — | 24 |
| 24 | 30 | 32 | — | 28 |

[a]P = cisplatin and/or carboplatin; A = doxorubicin; C = cyclophosphamide. [b]Cyclophosphamide dose-response is negative for median survival.

The methods of this paper do not solve the concerns about assumptions of the dose intensity calculation, particularly in regards to multidrug regimens (Gelman, 1990; Gelman & Neuberg, 1991). Limitations of the design of the clinical trials employed made it impossible to evaluate synergistic effects or schedule dependencies. There are other issues that can also influence the interpretation of our results. Firstly, we used only published data and hence results could be affected by publication bias (Begg & Berlin, 1989), which could be further accentuated by the fact that only between half and two-thirds of the published studies had enough data for inclusion in this study. Secondly, median survival may not be the most sensitive endpoint for measuring the impact of

**Figure 4** Partial regression plots for relative median survival *vs* adjusted relative cisplatin dose (left panel), doxorubicin dose (middle panel), and cyclophosphamide dose (right panel). Plots represent the increase in median survival for a change in dose intensity within any given study holding the doses of the other agents fixed. Vertical axes are on log scales.

dose intensity. Unfortunately, long term survival, perhaps a more sensitive endpoint, was not available in many studies. Moreover, it was not possible to evaluate the effect of the subsequent management on survival. This could partially account for the less impressive relationship between dose-intensity and survival compared to dose-intensity and response. Patients failing first-line non-platinum chemotherapy are more likely to respond to second-line treatment with platinum than are patients failing first-line platinum who receive other second-line treatments including second-line platinum. This observation has been used to partially explain the lack of survival benefit in randomised trials comparing platinum compounds with a single alkylating agent (Advanced Ovarian Cancer Trialist Group, 1991). Thirdly, we can estimate the relationship between a specified increase in total dose intensity and outcome (response and survival), but we cannot really estimate the shape of the relationship. Our estimates were based on the assumption of linearity, but the data were not sufficient to distinguish different shapes while controlling for study differences.

Nevertheless, the results presented here confirm that there is a relationship between overall dose intensity and response or survival after adjusting for study effects. For survival, this relationship seems smaller than the one found by Levin and Hryniuk (1987*a*). The units of dose intensity for individual drugs are defined by Table II and an increase in relative dose intensity of two units generally is accompanied by a major increase in serious or life threatening toxicities. It also has to be considered that there were few studies in which arms differed by more than one unit of total dose-intensity, and, consequently, it is not possible to make any comment on whether the relationship between outcome and dose-intensity continue beyond that range. This less optimistic result concerning dose-intensity and survival is in better agreement with the disappointing situations in advanced disease, but also indicates that the development of effective toxicity reduction agents would be of benefit in this chemosensitive neoplasm. The fact that the regression coefficients for survival are about the same for cisplatin and doxorubicin confirms the importance of using these drugs at an intensive rate. In addition, these results confirm the role of cisplatin in advanced ovarian carcinoma, in agreement with the AOCTG meta-analysis (Advanced Ovarian Cancer Trialist Group, 1991). They also underline the important role of doxorubicin, helping in the interpretation of the finding of a recent meta-analysis (Ovarian Cancer Meta-Analysis Project, 1991), which showed a superiority of a CAP over a CP regimen.

In summary, the validity of the dose intensity hypothesis in advanced ovarian cancer is substantiated based on the utilisation of improved methodology for analysis of available data. This approach suggests hypotheses for the intensification of therapy and reinforces the importance of formally evaluating dose intense regimens in prospective randomised clinical trials.

### References

ADVANCED OVARIAN CANCER TRIALIST GROUP (1991). Chemotherapy in advanced ovarian cancer: an overview of randomised clinical trials. *Br. Med. J.*, **303**, 884–893.

AMERICAN CANCER SOCIETY (1986). *Cancer Facts and Figures*. New York.

BEGG, C.B. & BERLIN, J.A. (1989). Publication bias and dissemination of clinical research. *J. Natl Cancer Inst.*, **81**, 107–115.

BONADONNA, G. & VALGUSSA, P. (1981). Dose response effect of adjuvant chemotherapy in breast cancer. *N. Engl. J. Med.*, **304**, 10–15.

DE VITA, V.T. (1986). Dose response is alive and well. *J. Clin. Oncol.*, **4**, 1157–1159.

GELLER, N.L., HAKES, T.B., PETRONI, G.R., CURRIE, V. & KAUFMAN, R. (1990). Association of disease-free survival and percent of ideal dose in adjuvant breast chemotherapy. *Cancer*, **66**, 1678–1684.

GELMAN, R. (1990). Keeping an open mind about the doses of chemotherapy. *J. Natl Cancer Inst.*, **82**, 1446–1447.

GELMAN, R. & NEUBERG, D. (1991). Making cocktails versus making soup. *J. Clin. Oncol.*, **9**, 200–203.

GREEN, J.A., DAWSON, A.A. & FELL, L.E. (1980). Measurement of drug dosage intensity in MVPP therapy in Hodgkin's disease. *Br. J. Clin. Pharm.*, **9**, 511–514.

HENDERSON, I.C., HAYES, D.F. & GELMAN, R. (1988). Dose response in the treatment of breast cancer: a critical review. *J. Clin. Oncol.*, **6**, 1501–1515.

JOHNSSON, J.E., TROPE, C., MATTSSON, W., GRUNSELL, H. & KLONYVES, I. (1979). Phase II study of LEO 1031 (Prednimustine in advanced ovarian carcinoma). *Cancer Treat. Rep.*, **63**, 421–423.

L'ABBE, K., DETSKY, A. & O'ROURKE, K. (1987). Meta-analysis in clinical research. *Ann. Int. Med.*, **107**, 224–233.

LASA, R.J., MURRAY, N. & COLDMAN, A.J. (1991). Dose intensity meta-analysis of chemotherapy regimens in small-cell carcinoma of the lung. *J. Clin. Oncol.*, **9**, 449–508.

LEVIN, L. & HRYNIUK, W.M. (1987*a*). Dose intensity analysis of chemotherapy regimen in ovarian carcinoma. *J. Clin. Oncol.*, **5**, 756–767.

LEVIN, L. & HRYNIUK, W.M. (1987*b*). The application of dose intensity problems in chemotherapy of ovarian and endometrial cancer. *Sem. Oncol.*, **14** (Suppl. 4), 12–19.

MANGIONI, C., BOLIS, G., PECORELLI, S., BRAGMAN, K., EPIS, A. & FAVALLI, G. (1989). Randomized trial in advanced ovarian cancer comparing cisplatin and carboplatin. *J. Natl Cancer Inst.*, **19**, 1464–1471.

MEYER, R.M., HRYNIUK, W.M. & GOODYEAR, M.D.E. (1991). The role of dose intensity in determining outcome in intermediate-grade Non-Hodgkin's lymphoma. *J. Clin. Oncol.*, **9**, 339–347.

OVARIAN CANCER META-ANALYSIS PROJECT (1991). Cyclophosphamide plus cisplatin versus cyclophosphamide, doxorubicin, and cisplatin chemotherapy of ovarian carcinoma: a meta-analysis. *J. Clin. Oncol.*, **9**, 1668–1674.

RICHARDSON, G.S., SKULLY, R.E. & NELSON, J.H. Jr (1985). Common epithelial cancer of the ovary. *N. Engl. J. Med.*, **312**, 415–424.

VELLEMAN, P.F. & WELSCH, R.E. (1981). Efficient computing of regression diagnostics. *The Amer. Statist.*, **35**, 234–242.

YOUNG, R.C., HUBBARD, S.P. & DE VITA, V. (1974). The chemotherapy of ovarian carcinoma. *Cancer Treat. Rep.*, **1**, 99–110.

### Appendix 1

List of 33 studies used for the analysis

BARLOW, J.J. & PIVER, M.S. (1977). Single agent *vs* combination chemotherapy in the treatment of ovarian cancer. *Obst. Gyne.*, **49**, 609–611.

YOUNG, R.C., CHABNER, B.A., HUBBARD, S.P., *et al* (1978). Advanced ovarian adenocarcinoma – a prospective clinical trial of melphalan (L-PAM) versus combination chemotherapy. *NEJM*, **299**, 1261–1266.

EDMONSON, J.H., FLEMING, T.R., DECKER, D.G. *et al* (1979). Different chemotherapeutic sensitivities and host factors affecting prognosis in advanced ovarian carcinoma versus minimal residual disease. *Cancer Treat. Rep.*, **63**, 241–247.

BARLOW, J.J., PIVER, M.S. & LELE, S.B. (1980). High-dose methotrexate with 'RESCUE' plus cyclophosphamide as initial chemotherapy in ovarian adenocarcinoma – a randomized trial with observations on the influence of C parvum immunotherapy. *Cancer*, **46**, 1333–1338.

MEDICAL RESEARCH COUNCIL'S WORKING PARTY ON OVARIAN CANCER: MEDICAL RESEARCH COUNCIL STUDY ON CHEMOTHERAPY IN ADVANCED OVARIAN CANCER (1981). *Br. J. Obst. & Gyne.*, **88**, 1174–1185.

BRUCKNER, H.W., COHEN, C.J., GOLDBERG, J.D., *et al* (1981). Improved chemotherapy for ovarian cancer with cis-diamminedichloroplatinum and adriamycin. *Cancer*, **47**, 228?–2294.

CARMO-PEREIRA, J., OLIVEIRA COSTA, F., HENRIQUES, E. & ALMEIDA R.J. (1981). Advanced ovarian carcinoma: a prospective and randomized clinical trial of cyclophosphamide versus combined chemotherapy (Hexa-CAF). *Cancer*, **48**, 1947–1951.

SCHWARTZ, P.E., LAWRENCE, R. & KATZ, M. (1981). Combination chemotherapy for advanced ovarian cancer: a prospective randomized trial comparing hexamethylmelamine and cyclophosphamide to doxorubicin and cyclophosphamide. *Cancer Treat. Rep.*, **65**, 137–141.

BELL, D.R., WOODS, R.L., LEVI, J.A. *et al* (1982). Advanced ovarian cancer: a prospective randomized trial of chlorambucil versus combined cyclophosphamide and cis-diamminedichloroplatinum. *Austr. N.Z. J. Med.*, **12**, 245–249.

OMURA, G.A., MORROW, C.P., BLESSING, J.A., *et al* (1983). A randomized comparison of melphalan versus melphalan plus hexamethylmelamine versus adriamycin plus cyclophosphamide in advanced ovarian carcinoma. *Cancer*, **51**, 783–789.

CARMO-PEREIRA, J., OLIVEIRA-COSTA, F. & HENRIQUES, E. (1983). Cis-platinum, adriamycin, and hexamethylmelamine versus cyclophosphamide in advanced ovarian carcinoma. *Cancer Chemother. & Pharmacol.*, **10**, 100–103.

EDWARDS, C.L., HERSON, J., GERSHENSON, D.M., *et al* (1983). A prospective randomized clinical trial of melphalan and cisplatinum versus hexamethylmelamine, adriamycin and cyclophosphamide in advanced ovarian cancer. *Gyne. Onc.*, **15**, 261–277.

NEIJT, J.P., VAN DER BERG, M.E.L., VRIESENDORP, R., *et al* (1984). Randomized trial comparing two combination chemotherapy regimens (hexa-CAF *vs* Chap-5) in advanced ovarian carcinoma. *Lancet*, **ii**, 594–600.

LAMBERT, H.E. & BERRY, R.J. (1985). High-dose cisplatin compared with high-dose cyclophosphamide in the management of advanced epithelial ovarian cancer (FIGO stages III and IV): report from the North Thames Cooperative Group. *Brit. Med. J.*, **290**, 889–893.

BRUCKNER, H.W., DINSE, G.E., DAVIS, T.E., *et al* (1985). A randomized comparison of cyclophosphamide, adriamycin, and 5-fluorouracil with triethylenethiophosphoramide and methotrexate. Both as sequential and as fixed rotational treatment in patients with advanced ovarian cancer. *Cancer*, **55**, 26–40.

SESSA, C., BOLIS, G., COLOMBO, N., *et al* (1985). Hexamethylmelamine, adriamycin and cyclophosphamide (HAC) versus Cis-dichlorodiamineplatinum, adriamycin and cyclophosphamide (PAC) in advanced ovarian cancer: a randomized clinical trial. *Cancer Chemother. & Pharmacol.*, **14**, 222–228.

EDMONSON, J.H., MCCORMACK, G.W., FLEMING, T.R., *et al* (1985). Comparison of cyclophosphamide plus cisplatin versus hexamethylmelamine, cyclophosphamide, doxorubicin and cisplatin in combination as initial chemotherapy for stage III and IV ovarian carcinomas. *Cancer Treat. Rep.*, **69**, 1243–1248.

AABO, K., HALD, I., HORBOV, S., *et al* (1985). A randomized study of single agent *vs* combination chemotherapy in FIGO staged IIb, III and IV ovarian adenocarcinoma. *Eur. J. Cancer Clin. Oncol.*, **21**, 475–481.

WILLIAMS, C.J., MEAD, G.M., MACBETH, F.R., *et al* (1985). Cisplatin combination chemotherapy versus chlorambucil in advanced ovarian carcinoma: mature results of a randomized trial. *J. Clin. Oncol.*, **3**, 1455–1562.

OMURA, G., BLESSING, J.A., EHRLICH, C.E., *et al* (1986). A randomized trial of cyclophosphamide and doxorubicin with or without cisplatin in advanced ovarian carcinoma. *Cancer*, **57**, 1725–1730.

GYNAECOLOGICAL GROUP CLINICAL ONCOLOGICAL SOCIETY OF AUSTRALIA AND THE SYDNEY BRANCH, LUDWIG INSTITUTE FOR CANCER RESEARCH (1986). Chemotherapy of advanced ovarian adenocarcinoma: a randomized comparison of combination versus sequential therapy using chlorambucil and cisplatin. *Gyne. Oncol.*, **23**, 1–13.

CONTE, P.F., BRUZZONE, M., CHIARA, S., *et al* (1986). A randomized trial comparing cisplatin plus cyclophosphamide verus cisplatin, doxorubicin and cyclophosphamide in advanced ovarian cancer. *J. Clin. Oncol.*, **4**, 965–971.

BERTELSEN, K., JAKOBSEN, A., ANDERSEN, J.E., et al (1987). A randomized study of cyclophosphamide and cis-platinum with or without doxorubicin in advanced ovarian carcinoma. *Gyne. Oncol.*, **28**, 161–169.

ADAMS, M., JOHANSEN, K.A., JAMES, K.W., *et al* (1982). A controlled clinical trial in advanced ovarian cancer. *Clin. Radiol.*, **33**, 161–163.

NEIJT, J.P., TEN BOKKEL HUININK, W.W., VAN DER BURG, M.E.L., *et al* (1987). Randomized trial comparing two combination chemotherapy regimens (CHAP-5 *v* CP) in advanced ovarian carcinoma. *J. Clin. Oncol.*, **5**, 1157–1168.

GRUPPO INTEREGIONALE COOPERATIVO ONCOLOGICO GINECOLOGIA (1987). Randomized comparison of cisplatin with cyclophosphamide/cisplatin and with cyclophosphamide/doxorubicin/cisplatin in advanced ovarian cancer. *Lancet*, **ii**, 353–359.

CLAES TROPE (1987). Melphalan with and without doxorubicin in advanced ovarian cancer. *Obst. & Gyne.*, **70**, 582–586.

HERNADI, Z., JUHASZ, B., POKA, R., *et al* (1988). Randomized trial comparing combinations of cyclophosphamide and cisplatin without or with doxorubicin or 4-epi-doxorubicin in the treatment of advanced ovarian cancer. *Int. J. Gyne. Obst.*, **27**, 199–204.

TOMIROTTI, M., PERRONE, S., *et al* (1988). Cisplatin (P) verus cyclophosphamide, adriamycin and cisplatin (CAP) for stage III–IV epithelial ovarian carcinoma: a prospective randomized trial. *Tumori*, **74**, 573–577.

LEONARD, R.C.F., SMART, G.E., LIVINGSTONE, J.R.B., *et al* (1989). Randomized trial comparing prednimustine with combination chemotherapy in advanced ovarian carcinoma. *Cancer Chemother. & Pharmacol.*, **23**, 105–110.

OMURA, G.A., BUNDY, B.N., BEREK, J.S., *et al* (1989). Randomized trial of cyclophosphamide plus cisplatin with or without doxorubicin in ovarian carcinoma: a gynecologic oncology group study. *J. Clin. Oncol*, **7**, 457–465.

MANGIONI, C., BOLIS, G., PECORELLI, S. *et al* (1989). Randomized trial in advanced ovarian cancer comparing cisplatin and carboplatin. *JNCI*, **81**, 1464–1471.

ADAMS, M., KERBY, I.J., ROCKER, I., *et al* (1989). A comparison of the toxicity and efficacy of cisplatin and carboplatin in advanced ovarian cancer. *Acta Oncol.*, **28**, 57–60.

**List studies not evaluable for the analysis**

DECKER, D.G., FLEMING, T.R., MALKASIAN, G.D., *et al* (1982). Cyclophosphamide plus cis-platinum in combination: treatment program for stage III or IV ovarian carcinoma. *Obst. & Gyne.*, **60**, 481–487.

GRONROOS, M., NIEMIEN, U., KAUPPILA, A., *et al* (1984). A prospective randomized national trial for treatment of ovarian cancer: the role of chemotherapy and external irradiation. *Eur. J. Obst. Gyne. Reprod. Biol.*, **17**, 33–42.

WILBUR, D.W., RENTSCHLER, R.E., WAGNER, R.J., *et al* (1987). Randomized trial of the addition of cis-platin (DDP) and/or BCG to cyclophosphamide (CTX) chemotherapy for ovarian carcinoma. *J. Surg. Oncol.*, **34**, 165–169.

SCHWARTZ, P.E., CHAMBERS, J.T., KOHORN, E.I., *et al* (1989). Tamoxifen in combination with cytotoxic chemotherapy in advanced epithelial ovarian cancer – a prospective randomized trial. *Cancer*, **63**, 1074–1078.

EDMONSON, J.H., MCCORMACK, G.M., WIEAND, H.S., *et al* (1989). Cyclophosphamide-cisplatin versus cyclophosphamide-carboplatin in stage III–IV ovarian carcinoma: a comparison of equally myelosuppressive regimens. *JNCI*, **81**, 5000–2504.

DEPALO, G.M., DELENA, M., BONADONNA, G., *et al* (1977). Adriamycin versus adriamycin plus melphalan in advanced ovarian carcinoma. *Cancer Treat. Rep.*, **61**, 355–357.

DEPALO, G.M., DELENA, M., DIRE, F., *et al* (1975). Melphalan versus adriamycin in the treatment of advanced carcinoma of the ovary. *Surg. Gyne. & Obst.*, **141**, 899–902.

BOLIS, G., BORTOLOZZI, G., CARINELLI, G., *et al* (1980). Low-dose cyclophosphamide versus adriamycin plus cyclophosphamide in advanced ovarian cancer – a randomized clinical study. *Cancer Chemother. & Pharmacol.*, **4**, 129–132.

BARKER, G.H. & WILTSHAW, E. (1981). Randomized trial comparing low-dose cisplatin and chlorambucil with low-dose cisplatin, chlorambucil and doxorubicin in advanced ovarian carcinoma. *Lancet*, **i**, 747–750.

BARLOW, J.J., SHASHIKANT, B. & LELE, S.B. (1984). Cisplatin-MECY (methotrexate-leucoverin rescue plan cyclophosphamide) versus cisplatin-CHAD (cyclophosphamide, hexamethylmelamine, doxorubicin and cisplatin) as initial chemotherapy in stage III–IV ovarian adenocarcinoma. *Cancer Treat. Rep.*, **68**, 1433–1438.

DELGADO, G., SMITH, F.P., MCLAUGHLIN, E.K. & TUKOLSKY, N. (1985). Single agent combination chemotherapy for ovarian cancer. *Amer. J. Clin. Oncol.*, **8**, 33–37.

WILTSHAW, E., EVANS, B., RUSTIN, G., *et al* (1986). A prospective randomized trial comparing high-dose cisplatin with low-dose cisplatin and chlorambucil in advanced ovarian carcinoma. *J. Clin. Oncol.*, **4**, 722–729.

BEZWODA, W.R. (1986). Treatment of advanced ovarian cancer: a randomized trial comparing adriamycin or 4-epi-adriamycin in combination with cisplatin and cyclophosphamide. *Med. & Ped. Oncol.*, **14**, 26–29.

ALBERTS, D.S., MASON-LIDDIL, N., O'TOOLE, R.V., *et al* (1989). Randomized phase III trial of chemotherapy in patients with previously untreated stages III and IV suboptimal disease ovarian cancer: a Southwest Oncology Group Study. *Gyne. Oncol.*, **32**, 8–15.